# Supplementary material for: The Modifying Role of Socioeconomic Position and Greenness on the Short-Term Effect of Heat and Air Pollution on Preterm Births in Rome, 2001–2013
Source: Int J Environ Res Public Health. 2019 Jul 12;16(14):2497. doi: 10.3390/ijerph16142497 (PMC6678295; doi:10.3390/ijerph16142497)
Supplement: Supplementary file 1 [file ijerph-16-02497-s001.pdf]

Table S1. Pearson's Correlation coefficients among the studied exposures.

	Daily maximum apparent temperature (°C)	PM_{10} (μg/m^3)	Ozone (μg/m^3)	NO_2 (μg/m^3)
Daily maximum apparent temperature (°C)	1			
PM_{10} (μg/m^3)	0.27	1		
Ozone (μg/m^3)	0.56	0.11	1	
NO_2 (μg/m^3)	-0.12	0.48	-0.09	1

Table S2. Poisson regression estimated percent change in daily number of preterm births by 1°C increase of MAT (Lag 0-2) for residential proximity to green spaces and SEP; Rome, 2001-2013.

**daily MAT (Lag 0-2)**		**SEP**		
		**High**	**Medium**	**Low**
**Residential proximity to green spaces**	**within 100m**	2.20 (0.22;4.21)	2.60 (-0.19;5.51)	5.28 (2.97;7.65)
	**between 100 - 500m**	1.10 (-0.23;2.44)	2.60 (0.79;4.44)	2.57 (1.10;4.05)
	**beyond 500m**	-0.10 (-1.72;1.69)	2.11 (0.13;4.13)	1.27 (-0.31;2.87)

Table S3. Poisson regression estimated percent change in daily number of preterm births by 1 °C increase of MAT (Lag 0-2) for residential proximity to green spaces and maternal age; Rome, 2001-2013.

**daily MAT (Lag 0-2)**		**Maternal Age**		
		**<30 yrs**	**30-36 yrs**	**>=37 yrs**
**Residential proximity to green spaces**	**within 100m**	3.12 (0.55;5.76)	4.42 (2.47;6.42)	1.30 (-1.23;3.90)
	**between 100 - 500m**	3.12 (1.38;4.89)	1.65 (0.35;2.98)	1.38 (-0.17;2.95)
	**beyond 500m**	1.97 (-0.02;4.00)	1.53 (0.03;3.07)	-0.57 (-2.38;1.27)
